# Potential effects of incorporating fertility control into typical culling regimes in wild pig populations

**DOI:** 10.1371/journal.pone.0183441

**Published:** 2017-08-24

**Authors:** Kim M. Pepin, Amy J. Davis, Fred L. Cunningham, Kurt C. VerCauteren, Doug C. Eckery

**Affiliations:** 1 National Wildlife Research Center, USDA, APHIS, Wildlife Services, Fort Collins, Colorado, United States of America; 2 National Wildlife Research Center, USDA, APHIS, Wildlife Services, Mississippi State, United States of America; Sichuan University, CHINA

## Abstract

Effective management of widespread invasive species such as wild pigs (*Sus scrofa*) is limited by resources available to devote to the effort. Better insight of the effectiveness of different management strategies on population dynamics is important for guiding decisions of resource allocation over space and time. Using a dynamic population model, we quantified effects of culling intensities and time between culling events on population dynamics of wild pigs in the USA using empirical culling patterns and data-based demographic parameters. In simulated populations closed to immigration, substantial population declines (50–100%) occurred within 4 years when 20–60% of the population was culled annually, but when immigration from surrounding areas occurred, there was a maximum of 50% reduction, even with the maximum culling intensity of 60%. Incorporating hypothetical levels of fertility control with realistic culling intensities was most effective in reducing populations when they were closed to immigration and when intrinsic population growth rate was too high (> = 1.78) to be controlled by culling alone. However, substantial benefits from fertility control used in conjunction with culling may only occur over a narrow range of net population growth rates (i.e., where net is the result of intrinsic growth rates and culling) that varies depending on intrinsic population growth rate. The management implications are that the decision to use fertility control in conjunction with culling should rely on concurrent consideration of achievable culling intensity, underlying demographic parameters, and costs of culling and fertility control. The addition of fertility control reduced abundance substantially more than culling alone, however the effects of fertility control were weaker than in populations without immigration. Because these populations were not being reduced substantially by culling alone, fertility control could be an especially helpful enhancement to culling for reducing abundance to target levels in areas where immigration can’t be prevented.

## Introduction

Whether native or introduced, wild pigs (or feral swine; *Sus scrofa*) threaten livestock health, human health and agricultural productivity in many countries across the globe [1–5]. Most countries have some level of population management to counteract their fecundity, damage to agriculture, and disease threats. Despite continued lethal control of wild pig populations in the USA [5–8], populations have continued to grow and expand geographically in many areas [9], with a current estimate of over 6 million pigs [7,10]. To manage population growth and reduce damage from invasive wild pigs in the USA, the National Feral Swine Damage Management Program (NFSDMP) has been implemented by the United States Department of Agriculture (USDA) Animal and Plant Health Inspection Service (APHIS), Wildlife Services (WS). The goal of the program is to eradicate wild pigs in many states eventually, and minimize their damage in states where eradication is infeasible.

Previous successful control programs in the USA have used a variety of population management techniques [11–14]. The most commonly used techniques for lethal control of wild pigs include trapping, ground shooting and aerial gunning. Registered sterilants for wild pigs do not yet exist for use in the USA. Efficacy of available techniques depends on population density, environment type (e.g. grasslands versus heavily wooded), weather and season [11,15–18]. Also techniques often differ in their temporal frequency of application due to inherent logistical differences among them. For example, aerial gunning is expensive by the hour and requires substantial planning to implement, whereas trapping and ground shooting are labor intensive and it is difficult to cover a large spatial area as quickly as aerial gunning. Therefore, effective trapping and ground shooting activities usually involve culling of fewer individuals per unit time [8,13,19] relative to aerial gunning, which is often characterized by removing many individuals sporadically [14,18]. Much progress has been made with technological development and effective application of commonly used control tools [5,15,20–22]. Similarly, previous wild pig management efforts in the USA [8,11–14] have provided valuable insight into the time and expense needed to reduce or eradicate local populations in specific areas. Less well understood are the general effects of management activities on population dynamics, which is important for planning resource allocation in large-scale eradication programs [13,23,24] that span a variety of different demographic conditions.

The large scale of the NFSDMP poses a substantial challenge. There are fewer dollars per km^2^ per year for control operations relative to local-scale eradication programs [13], and there are not enough resources to cover all high-damage areas simultaneously. Within states that have very high pig abundances a common strategy is to respond to high-damage areas with a short, intense culling effort in a small geographic area, then reposition resources to other priority sites, and return when damage is high again. In these cases, the population could rebound quickly to levels causing high-damage [25] rather than giving a long damage-reduced period. An unanswered question for strategic planning in high-abundance states is: At what frequency and culling intensity do wild pigs need to be culled from local areas in order to decrease their population growth rates to target levels? The answer will depend largely on demographic dynamics of the population. Thus, in order to develop plans for optimizing allocation of fiscal and human resources broadly across the country, it is important to improve our understanding of how the magnitude and frequency of culling affect population dynamics over a realistic range of wild-pig demographic conditions. Population models have provided insight on culling intensities required for reaching particular management goals [26–29] and conditions when fertility control may be effective [30–32]. Results range widely suggesting that culling intensities of 10–65% annually are needed to cause populations declines. Some of this uncertainty is likely due to different levels of model complexity (i.e., Leslie matrix deterministic models versus stochastic individual-based models), which account for different amounts of biological realism. Another potential reason likely comes from using demographic parameters from different geographic regions (i.e., Australia; Hawaii, USA; Tennesee, USA; California, USA).

In addition to culling, research by USDA-APHIS-WS and the broader scientific community is being conducted to identify new tools for enhancing population control work, including sterilants [33]. The utility of fertility control in large-scale culling programs is much debated, but this type of contraception-based tool is being discussed and researched with the goal of potentially applying it as a complement to existing culling programs in wild pig populations. Because an efficient, registered sterilant for wild pigs requires much time and expense to develop and register, it is important to carefully consider its potential benefits preceding investment. Theoretical work has shown that fertility control is most effective when applied in conjunction with culling [30–32]. However, the efficacy of fertility control depends on intrinsic population growth rate [31], emphasizing that potential outcomes must be studied using population biology of the target species under a variety of possible demographic conditions. In addition to intrinsic population growth rate, an important factor affecting the efficacy of fertility and culling programs is immigration from surrounding unmanaged populations [32,34–37]. In large-scale wildlife management programs, immigration is likely to be an important factor because not all areas can be controlled simultaneously. However, our current understanding of the effects of realistic culling patterns on wild pig population dynamics in the USA is weak, and even poorer is our ability to predict how demographic conditions such as fertility and immigration affect management outcomes, which impedes science-based planning of control work.

Wild pigs are a social species. Populations are typically organized in matrilineal groups of adult females with their young, and small groups (2–3 individuals) of sub-adult males or solitary adult males [38]. Family groups are ever-changing in terms of size and composition; individuals may leave to join other groups [38]. Territoriality of groups and solitary males could act to regulate density on the landscape to some degree [38–40]. Monthly and overall home range sizes are small (mean = 3.4 km^2^, standard deviation = 4.6; mean = 6.1 km^2^, standard deviation = 7.8), and average daily movement is rarely beyond 0.35 km (standard deviation = 0.34) [41]. Natal dispersal occurs primarily in males [38]. Males may disperse in small groups initially but are almost always solitary after 3 years of age [38,42]. This complex spatial ecology likely plays an important role in shaping density across the landscape and population response to control. In fact, some wild pig control techniques, for example corral trapping, have been designed to target family groups as they have the highest reproductive potential. Thus accounting for individual-level variation in wild pig spatial ecology could be important for predicting the effects of control on wild pig populations.

To address knowledge gaps and quantify the effects of wild pig control in the USA, we examined the effects of culling patterns employed by USDA-APHIS-WS. We compared three models of varying complexity to understand how different modeling assumptions may affect predictions of management outcomes: 1) a spatially-explicit individual-based model parameterized with field-based measures of demographic processes, 2) an age-structured, population-level deterministic model with demographic parameters scaled to match population *growth* rates in the individual based model, and 3) a logistic model with demographic parameters scaled to match population growth rates in the individual based model. Because there is uncertainty associated with pig population dynamics, we examined the effects of culling patterns using a range of demographic conditions (i.e., birth rates, average dispersal distance, maximum family group size, presence of immigration from neighboring populations) in the individual-based model. We also identified conditions where fertility control could substantially accelerate population decline.

## Methods

**Fig 1:**
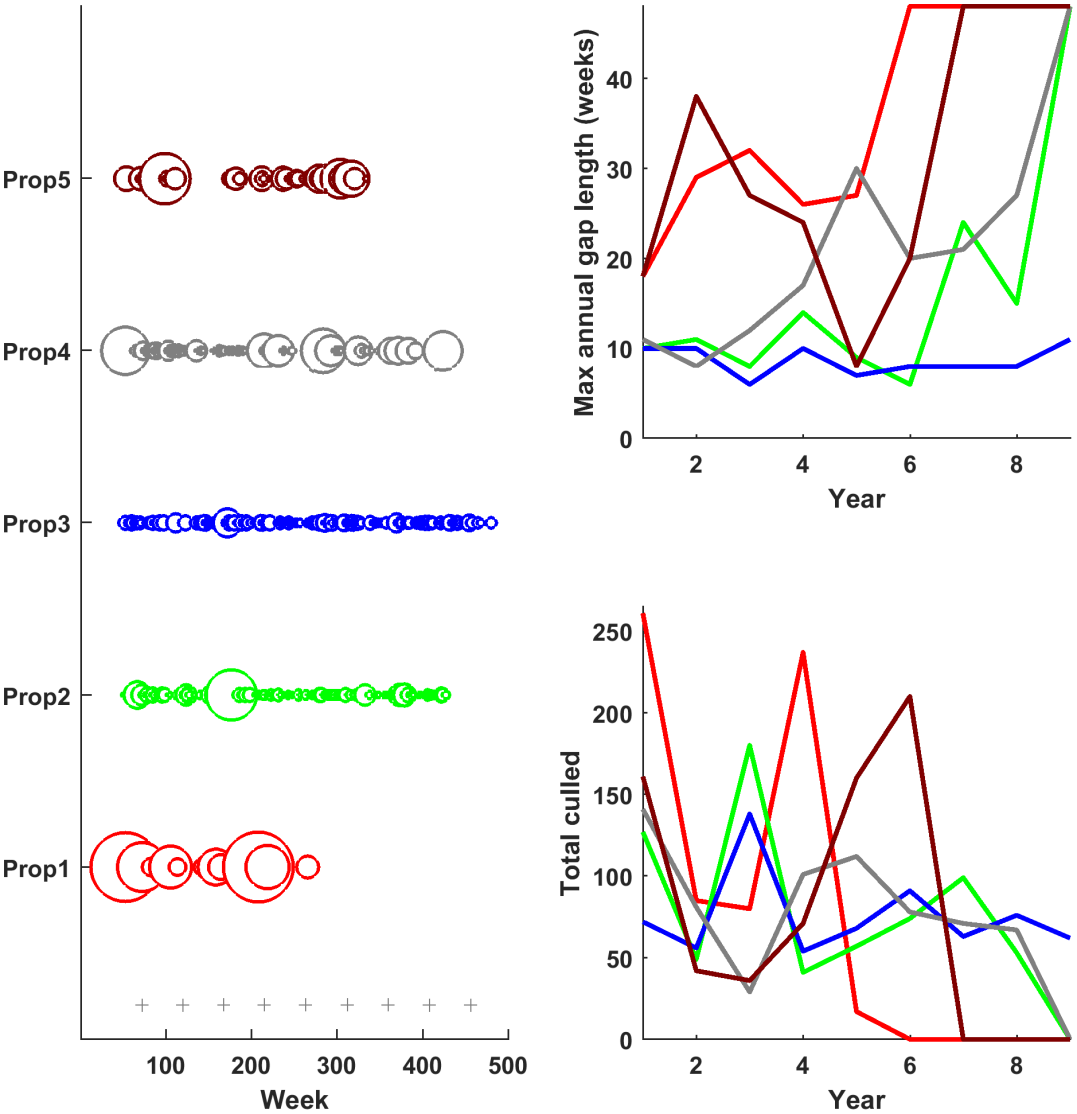
Five culling patterns. A. Number of wild pigs culled (size of the circles) during each day of population management (X-axis) according to 5 culling patterns (labeled 1 to 5 on Y-axis). The largest circle corresponds to 171 wild pigs, the smallest corresponds to 1. Grey plus signs along the bottom indicate the timing of contraceptive control (hypothetical), if applicable. B. The maximum number of weeks with no culling during the year. C. Total culled per year. Total pigs culled by each method was 680. Line colors in B and C correspond to culling pattern labels in A.

To examine the impact of different culling patterns on wild pig populations in the USA, we developed three population-dynamic models that incorporated varying levels of biological complexity. We implemented the individual-based and population-level logistic models in Matlab (Version R2016b, The MathWorks, Inc., Natick, Massachusetts, United States) and the age-structured population-level model in R (R Core Team, 2015, Vienna, Austria). We modeled small populations (~500 pigs) because individual management efforts most often operate in small geographic areas [12] and because we were interested in considering the stochastic effects of abundance as a population nears eradication. We obtained data on culling patterns of pigs from the USDA-APHIS-WS Management Information System (MIS) database. USDA-APHIS-WS provides wildlife control assistance to land owners based on the authority of the Animal Damage Control Program of 1985 in compliance with the National Environmental Policy Act. We selected five different properties that: 1) had long-term (> 5 years), persistent culling, 2) ranged from 8.5–40.5 km^2^ to represent areas that could be considered a single population, and 3) differed qualitatively in the temporal frequency and intensity of culling (i.e., showing different culling patterns; Fig 1). We applied the empirical culling data to simulated population dynamics to evaluate efficacies of the different patterns. We also investigated the effects of sterilizing a proportion of the population in addition to culling, to examine potential benefits of mixed management strategies (i.e., culling alone versus culling and fertility control). Below, we describe implementation of the stochastic individual-based model first, followed by the deterministic, discrete-time models.

### Stochastic individual-based model

#### Process overview

The individual-based model accounted for spatial, social and age-structure dynamics, which are all thought to impact efficacy of management. At the start of simulations, individuals were assigned several attributes that were monitored and updated at a weekly time step. Individual-level attributes included age in weeks, sex, unique group identification, dispersal distance, age of natural mortality, x coordinate, y coordinate, and grid cell ID. Thus, individuals were assigned to a particular home range centroid within a grid cell. Male-specific attributes included dispersal age. Female-specific attributes included age at which conception became possible (i.e., reproductive maturity, Table 1), reproductive status (fertile or not) and weeks pregnant (if gestating). Sex was fixed throughout life. Dispersal distance varied stochastically according to Poisson distribution (Table 1). Other attributes changed based on time, age, group size and grid-cell density. Parameters used to guide changes in attributes are listed in Table 1. Attributes were updated according to the following processes listed in order of occurrence (note that specific details of processes involved in each step are treated in more detail in the next section): 1) culling of individuals that reached age of natural mortality, 2) culling a fixed number of individuals nearest a random point based on each culling pattern, 3) sterilization of a proportion of reproductively active females selected at random (only for simulations involving sterilization), 4) dispersal to new home range centroids, 5) density-dependent mortality, 6) new conceptions by selecting reproductively-active, non-lactating females at random, 7) births: assignment of individual-level attributes to new litters, and 8) recording abundance and numbers culled. For simplicity with converting monthly empirical data, we assumed there were 4 weeks per month and thus each year included 48 weeks.

**Table 1:** Description of parameters.

Parameter	Values	References
**Age of natural mortality** (time before natural death occurs)	μ~EXP(3 years)	[68–70] (Table 6; pg 173 [7])
**Weekly conception probability per individual** (determines intrinsic population growth rate)	Monthly conception probabilities Jan.-Dec.: 0.1053, 0.0592, 0.0493, 0.0493, 0.0132, 0.0493, 0.0263, 0.1151, 0.2138, 0.1711, 0.0724, 0.0757; These values were divided by 4 and each value was replicated 4 times to convert from monthly to weekly conception probabilities; thus conception could occur during any month but was highest Aug.-Jan.	Estimated from: [7] Fig 1 pg 67
**Age-based scaling factor on conception probability** (age-based conception probability = conception probability x scaling factor)	0.5 (< 1 year); 0.75 (1–3 years); 1 (> 3 years)	[7]
**Overall scaling factor on conception probability** (manipulates intrinsic population growth rate: overall intrinsic population growth rate = age-based conception probability x overall scaling factor)	0.5 (low; λ = 1.3, *r* = 0.26); 1 (field-based; λ = 1.78, *r* = 0.58); 3 (high; λ = 2.43, *r* = 0.89)	
**Litter size** (number of viable offspring per litter)	3 piglets (< 1 year); 7 piglets (1–3 years); 10 piglets (> 3 years)	[51,71,72]
**Age at reproductive maturity (females only)** (minimum age that females may conceive)	6 months	[73] [74]
**Minimum time between farrowing and conception**	3 months	[75]
**Gestation time**	18 weeks	[76]
**Age of male dispersal from family group** (brothers initially disperse together and form a group of young males)	~POISSON(36 weeks)	[7,77,78]
**Age that young male groups dissolve and males become independent**	2 years	[42]
**Dispersal distance**	Variable; ~EXP(ξ); where ξ = 0.5, 1.5, or 3 km	[79]
**Maximum family group size** (carrying capacity for family groups; at carrying capacity some females will disperse and form new family groups)	Variable; 10, 20 or 30 pigs	[38]
**Initial age distribution** (proportion of individuals in each age class at the outset of simulations; following the initialization, population dynamics were allowed to occur for 10 years before beginning culling treatments)	0–1: 56.5%; 1–2: 16.2%; 2–3: 11.1%; 3–4: 7.5%; 4–5: 4.3%; 5–6: 2.4%; >6: 1.9%	[7] Table 3, pg 168

#### Social and spatial structure

Individuals were grouped based on age and sex characteristics as follows: 1) family groups (including females and pre-dispersal males), 2) small groups of young males (males less than two years old that have dispersed from family groups) or older males (greater than two years and occurring independently). Individuals in the same group were assigned the same random home range centroids. Home range centroids were selected at random within a grid cell. The full landscape was 100 km^2^, gridded at a 1 km x 1 km scale to be larger than daily movements but smaller than home range size, which includes dispersal distance [41]. This small scale was chosen because pig movements Culling only occurred in a central portion of the landscape, which was 25 km^2^ (Fig A in S1 File), and outputs were only recorded for this area. In runs without immigration, only the middle 25 km^2^ area was modeled. Grid cells each had a carrying capacity of 10 or 30 pigs, which controlled population density throughout the landscape. There were 2 patches of higher density environment type (30 pigs /km^2^) and 2 patches of lower density environment type (10 pigs /km^2^), for a maximum carrying capacity of 500 pigs over the middle 25 km^2^ (average density of 20 pigs /km^2^), which was the target for culling (Fig A in S1 File). When family groups grew beyond their maximum group capacity, the number of individuals beyond the maximum capacity dispersed to a new grid cell that had a total abundance below carrying capacity (one mechanism of density-dependent regulation; details described in *Dispersal* section). In addition, in grid cells where family groups were below the maximum group capacity but the total abundance was beyond the grid-cell carrying capacity, the population density was controlled by removing the number pigs beyond the grid-cell carrying capacity (density-dependent mortality–a second mechanism of density-dependent regulation). The youngest pigs were preferentially selected for removal consistent with density-dependent causes.

#### Dispersal

There were three types of permanent relocation for home range centroids of individuals (i.e., dispersal): natal dispersal (males only), adult male dispersal, female dispersal due to overcrowded family groups. For natal dispersal, males left the family group with their brothers, chosen at random from a Poisson distribution (Table 1) [42]. At 2 years of age, adult male dispersal occurred, where brother groups dissipated and all members became solitary adults [42]. Females remained with family groups unless family group size reached maximum capacity [38,43] (Table 1). For family groups above maximum group capacity, female dispersal due to overcrowded family groups occurred. The number of females beyond the maximum group capacity left the family group and formed a new family group with a distinct home range centroid. The number of females selected to leave were chosen at random with the restriction that they were older than the minimum natal dispersal age for males (fixed at 6 months). For all three types of dispersal, the dispersal algorithm proceeded the same as follows: 1) for each 45 degree angle from the home range centroid, a new possible set of [x,y] coordinates was obtained using a dispersal distance value assigned at random to the group (Table 1; i.e. x = distance x cos(angle) + current x coordinate, y = distance x sin(angle) + current y coordinate). If at least one of these eight potential locations were valid (i.e., valid = a grid cell with fewer pigs than the carrying capacity or a location off the grid), then a valid location was chosen at random and pig(s) were relocated there. If there were no valid locations, the distance value was doubled and the process repeated until a valid location was obtained. Thus, the dispersal algorithm acted in a density-dependent manner because pigs were not allowed to disperse to grid cells that were already at or above carrying capacity. In the runs with no immigration, pigs which emigrated from the culling area were lost permanently, whereas in the runs with immigration, pigs which emigrated from the culling area could relocate to land immediately outside the culling area and immigrate back at a later date when space became available. Pigs that relocated off the full grid were lost permanently. In the area outside the culling area, birth rates were much higher than emigration rates off the grid, such that there was a continuous ample supply of potential immigrants to the culling area.

#### Birth/death

For reproductive females, conceptions occurred randomly in reproductively active females according to a weekly conception probability (Table 1), which was the same for each month but varied across months based on an empirically-derived distribution of monthly conception probabilities (Table 1). We varied intrinsic population growth rate by multiplying the vector of weekly conception probability by a fixed scaling factor (Table 1 –possible values = 0.5, 1, or 3 referred to as ‘low’, ‘field-based’ and ‘high’ intrinsic population growth rate). When implementing these scaling factors in a population with no density-dependent regulation, a value of 1 (‘field-based’) generated a an average annual population growth rate (λ) of 1.78 ± 0.08—calculated as [∑_t_(N_t_/N_0_)]/T—where *t* = 1,…,T years (equivalent to intrinsic *r* = 0.26). Similarly, scaling values of 0.5 and 3 led to annual population growth rates of 1.3 ± 0.025 and 2.43 ± 0.35 (intrinsic *r* = 0.58 and 0.89), respectively. Our ‘low’ intrinsic population growth rate condition (λ = 1.3) is similar to values that have been estimated based on data from Texas, USA [44,45], however, that intrinsic population growth is likely higher as suggested by [46]. We included the high population growth (λ = 2.43) condition for comparison because wild pigs are known to be very fecund, and could potentially exhibit high population growth rates in parts of the USA where there is substantial introgression of domestic pig genes and abundant resources. The individual-based model approach does not allow for unrealistically high population growth because the number of births per female per year are limited by gestation births and lactational anestrus periods. After conception and a gestation period (Table 1), pregnant females farrowed litters with a male:female ratio of 1 [39,47–49]. Litter size (Table 1) and the maximum litters per year [50] both increased with age (Table 1); litter size was fixed within age classes (Table 1). After farrowing, females were unable to conceive again until after a lactational anestrus period (Table 1) and conception probability thereafter. Thus, the maximum number of litters per year was two [7,50]. We modeled natural mortality by assigning each individual an age of natural mortality at birth, which was an exponentially-distributed random number, such that the probability of living longer was smaller than the probability of dying young (Table 1). We modeled density-dependent mortality by removing a family group from each grid cell that reached the grid-cell specific carrying capacity. We culled entire groups, instead of a subset of individuals, to maintain realistic group structure and because we assumed that density-dependent effects would affect entire groups because young are dependent on their mothers. Births were not density-dependent [51].

#### Initial conditions

Populations were initialized as follows. Each individual was assigned an age, sex, dispersal age (males only) and age of natural mortality. Ages were chosen at random using an empirically-determined age-distribution [7] (Table 1). Sex was assigned at random according to a 1:1 ratio within each age class [39,47–49]. Dispersal age was chosen at random from an exponential distribution (Table 1). For males whose age was beyond dispersal age, dispersal status was recorded as completed. Age of natural mortality was chosen at random from an exponential distribution (μ, Table 1). Males older than 2 years were assigned NA for group ID. Males beyond dispersal age but less than 2 years were divided into groups of 5 (plus one smaller group of remaining individuals if applicable). Similarly, all females and males less than dispersal age were divided into group sizes that were ¼ of the maximum family group size (plus one smaller group of remaining individuals if applicable). Each individual or group was assigned to a grid cell ID chosen at random with replacement (the algorithm ensured that unoccupied grid cells were prioritized). Within each grid cell, the individual or group was given [x,y] coordinates selected at random. Other individual-level attributes were assigned as described in Table 1 based on age and sex. After the population was initialized, population dynamics occurred for 10 years (burn-in period), and the population matrix (defining size, distribution, and individual-level attributes) at the end of the 10 years was used as the starting point for all simulation conditions.

#### Culling

We trimmed the five culling patterns by removing events from the later end such that the total number of pigs culled in each pattern was consistent (680, the smallest number amongst the 5 patterns, S3 File). Thus, the main differences between the 5 patterns were in the frequency of culling events (i.e., gaps; Fig 1A and average number culled per event 3–50; Fig 1B) and culling intensity (mean number culled per year 85–165; Fig 1C). Throughout simulations of population dynamics, we culled a fixed number of wild pigs at specified time points, as determined from MIS data (Fig 1). We culled all individuals within the closest proximity of each other (using the home range centroid attribute) because wild pig culling techniques such as corral trapping and aerial gunning employ intense culling in focused areas and often target whole family groups when possible. For each set of demographic parameters, we also ran simulations with no culling as control populations. For all simulations we included a density-dependent capture probability (α) such that when density was low, not all the pigs targeted for capture were captured. Thus the realized number culled (*C*_t_) was:

$$\alpha =1-\left(\frac{1}{{\left(1+\beta \right)}^{Nt}}\right)$$ (1)

$$C_{t}=\alpha c_{t},$$ (2)

where β (scaling parameter on the relationship of pig density and capture probability) was fixed at 0.03 because it gave a relationship similar to [15], *N*_*t*_ was the abundance at time *t*, and *c*_*t*_ was the target number to cull.

#### Fertility control

In modeling fertility control, our goal was to examine its effects in concert with culling in a situation where lethal management strategies take priority over other strategies. We assumed the sterilant could be broadcast generally across the landscape (such as by aerial drop). Although such a product does not yet exist, our goal was to examine the potential effects of this type of ideal product. Sterilization impacts were simulated by randomly selecting a proportion of fertile (i.e., beyond the minimum age of conception) females to be sterilized (i.e., coverage) once per year (pattern of implementation in Fig 1A), chosen at random across the culling area. We assumed that sterility lasted 2 years and gestating individuals were sterilized but still gave birth to their current litter. We assessed a range of efficacies, by sterilizing a proportion (0.2–0.8) of reproductively active females at each event. For each new sterilization event, we did not distinguish between individuals that were already sterilized meaning that sterilized individuals could be part of the new proportion sterilized, but that the new event extended their current time period of infertility. We also assumed that sterilized individuals could be culled. Our assumptions were based on the logic that there is no easy way to distinguish sterilized from unsterilized individuals in the field.

#### Immigration

In order to examine effects of immigration we conducted simulations as above on larger landscapes (100 km^2^) where the target area of 25 km^2^ was in the center. Controls were only implemented within the 25 km^2^ target zone. Immigration occurred by dispersal (males at reproductive maturity or large family groups breaking apart to form separate groups) into the target area according to the parameters in Table 1 and described above.

### Sensitivity analyses

We conducted a sensitivity analysis on the following demographic parameters: maximum family group size, mean dispersal distance, and population growth rate (implemented through the scaling parameter on conception probability). We chose 3 values from each demographic condition and used a full factorial design (all possible combinations) for a total of 27 sets of demographic conditions (Table 1). For each set of demographic conditions, we ran the 5 different culling treatments and a scenario with no culling. For each culling/no culling scenario we tested 4 levels of sterilization (20, 40, 60 and 80% of the population). We also ran all conditions with and without immigration (i.e., dispersal of sub-adults from surrounding areas), making a total of 1458 sets of conditions. We ran 30 replicate simulations for each set of conditions (1458x30 = 43,740 simulations in total). All runs were conducted for 10 years.

### Statistical analyses

We used generalized linear models to interpret the effects of culling patterns and fertility control on population reduction. All analyses were implemented in Matlab using the Statistics toolbox. We summarized simulation output into 3 responses:

1. The average net annual population growth rate (net *r*) over the first 4 years of culling (Table A in S2 File). For this, we were interested in the effects of culling patterns (intensity and gap periods) and demographic conditions (intrinsic *r* and immigration status) on realized population growth rates. We calculated mean net *r* as: $net\;r=\;\frac{\sum_{t=1}^{T}log\left(\frac{N_{t}}{N_{0}}\right)}{T}$, where *T* = 4 years and *N* was from the last week of year *t*.
2. The minimum proportion of the population remaining after 4 years of culling (Table B in S2 File). For this we were interested in the same parameters as in 1) (except for gap period) on the magnitude to which the population could be reduced over 4 years. We calculated the proportion remaining as: N_t_/N_0_ where N_0_ was the initial abundance in the week before *any* culling began (i.e., last week of the burn-in period) and N_t_ was the abundance in the last week of year *t*, and t = 1,2,3, or 4. We then took the minimum value over the 4 years.
3. The difference in the proportion by which the population is reduced after 4 years (as calculated in 2)) in populations without and with fertility control. For this response, we were interested in the net *r* due to culling and coverage of fertility control, and the demographic effects of intrinsic *r* and immigration status.

**Fig 2:**
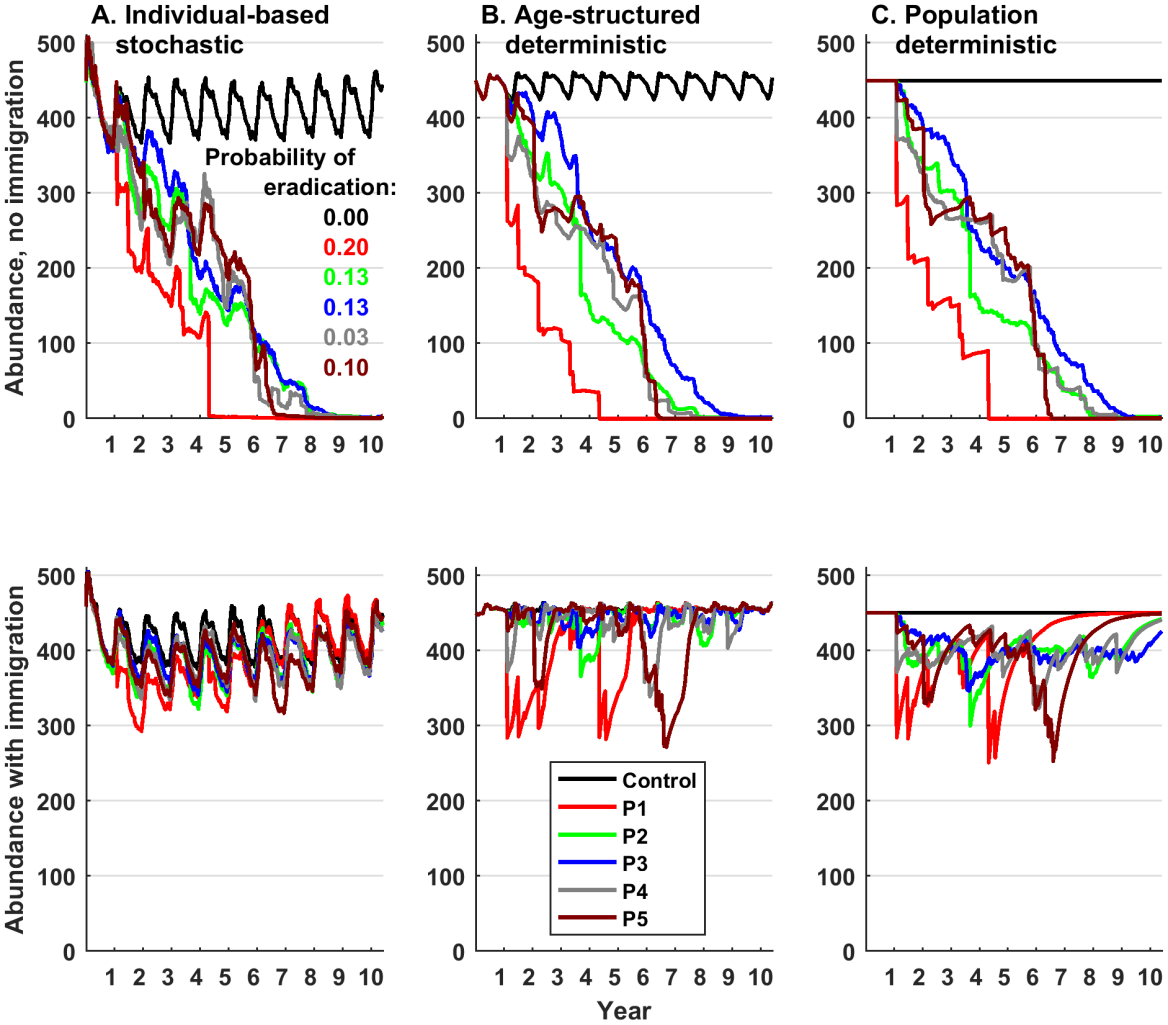
Effects of 5 culling patterns under different model structures. X-axis: weekly abundance over 10 years. Five different culling patterns are distinguished using the same color scheme as in Fig 1. Black lines indicate conditions with no culling. Top plots are for populations with no immigration; bottom plots are for populations with immigration. For the individual-based model, each line is the mean replicate simulations for runs that led to eradication within 10 years. The probability of eradication shows the proportion of 30 simulations that led to eradication. The mean behavior of runs that did not lead to eradication within 10 years is shown in Fig D in S1 File.

We chose 4 years as the time frame to examine effects because it is a reasonable amount of time to expect strong management outcomes and because plots of management effects over time (Fig 2) showed this to be the time frame to observe the strongest effects across the widest range of conditions (Fig B in S1 File). Details of statistical methods and estimated parameters are given in S2 File.

### Age-structured, population-level, deterministic model

We also used an age-structured, population-level, deterministic model to evaluate the advantage of including age-specific effects on demographic dynamics. We used discrete-time, age-based recursive equations as per Otto and Day [52] as follows:

$$N_{P,t+1}=\left(N_{P,t}*S_{P}*\left(\frac{23}{24}\right)+m_{P}\right)-\alpha c_{Pt}+b*\left(N_{J,t}*\;0.5*l_{J}+N_{Y,t}*\;0.75*l_{Y}+N_{A,t}*l_{A}\right)$$ (3)

$$N_{J,t+1}=\left(N_{J,t}*S_{J}*\left(\frac{23}{24}\right)+m_{J}+N_{P,t}*S_{P}*\left(\frac{1}{24}\right)\right)-\alpha c_{Jt}$$ (4)

$$N_{Y,t+1}=\left(N_{Y,t}*S_{Y}*\left(\frac{95}{96}\right)+m_{Y}+N_{J,t}*S_{J}*\left(\frac{1}{24}\right)\right)-\alpha c_{Yt}$$ (5)

$$N_{A,t+1}=\left(N_{A,t}*S_{A}+m_{A}+N_{Y,t}*S_{Y}*\left(\frac{1}{96}\right)\right)-\alpha c_{At}$$ (6)

Where the age classes are piglets (0–24 weeks of age), juveniles (25–48 weeks), sub-adults (49–144 weeks), and adults (145+ weeks). The discrete-time intervals are by week (*t*). The abundance is given by age class and week (e.g., *N*_*P*,*t*_). We calculated weekly survival probabilities (e.g., *S*_*P*,*t*_) by age class from the age of natural mortality distribution used in the individual-based model (Table 1). Both survival probability and immigration rates (*m*) are density dependent and thus are reduced if the total population (of all age classes *N*_*•*,*t*_) exceeds the carrying capacity (*K*). Since the weekly time scale is not the same as the timing of advancing to the next age class, we included a probability of ‘aging’ as the simple proportion of one in the number of weeks in that age class (e.g., 1/24 to age from juvenile to yearling and 1/48 to age from yearling to adult). The target culling patterns (*c*) and density-dependent capture process (α, Eq 1) are the same as described above. The distribution of age classes culled is the same as the proportion of each age class at a given time point. Birth probabilities (*b*) contribute to new piglets from the age classes that are of reproductive age (all put piglets). The conception probability used in the individual-based model was shifted by 4 months (gestation period) and used to model weekly birth probability. As in the individual-based model, the weekly birth probability was scaled by age class (0.5 for juveniles, 0.75 for sub-adults, and 1 for adults). Litter sizes (*l*) for the different age-classes were as in Table 1. In this model, all females are available every week to give birth. This is different from the individual-based model that assumes that gestation and lactating females are unavailable for conception. Thus, in order to capture the same population growth as the individual-based model (λ = 1.78), we used an additional overall scaling parameter on birth probability: 0.1426.

### Population-level, deterministic model

We also used a non-spatial, discrete-time logistic model with no social or age structure for comparison to the more complex individual-based model. The deterministic model was as follows:

$$N_{t}=\;\left(r_{t-1}N_{t-1}+\;\frac{m}{N_{t-1}}\right)\left(1-\frac{N_{t-1}}{K}\right)+\;N_{t-1}-\alpha c_{t-1}$$ (7)

N represents pig abundance, *t* is week, *r* is the intrinsic rate of increase (population growth rate), *m* is the immigration rate, *K* is the population carrying capacity, *c* is the target number of pigs to be culled (from empirical data described above), and α is the density-dependent capture probability (Eq 1). Once populations dropped below 1 they were considered eradicated. Parameter values were: conception probability vector (Table 1) times a scaling factor of 0.2183 (to match an annual population growth rate of λ = 1.78—the same as the individual-based model), *K* = 450 pigs, and *m* = *K* x 10. *m* included an arbitrary scaling factor, here 10, because there were no data to parametrize this value. We chose 10 because using this value, the dynamics were similar to our individual-based model. Note in this model population changes are instantaneous, whereas in the individual-based model population changes occur in bursts (i.e., litters) after a gestation period when females give birth.

## Results

Conception probability (which determined intrinsic population growth rate) had the strongest impact on population dynamics while maximum family group size and mean dispersal distance showed only marginal effects across the three parameters examined (Fig C in S1 File). Culling pattern 1 (Fig 1: Prop1) maintained the lowest wild pig abundances while culling was ongoing (Fig 2, Fig D in S1 File). This pattern was characterized by culling more pigs per year on average (Fig 1), and large numbers of pigs per event. Patterns 1, 4 and 5 all involved periods of no culling (up to ~ 1–2 years). For patterns 4 and 5 these gaps were enough to cease population decline, until culling was resumed later on (Fig 2). Also, during these gaps in culling, abundance for patterns 4 and 5 climbed above that in patterns 2 and 3.

In populations closed to immigration, all three models performed qualitatively similarly (Fig 2). The main difference was that both deterministic models predicted eradication within 10 years for all culling patterns whereas the stochastic model predicted relatively low eradication probability ranging from 0.03–0.20 (Fig 2A, top). In populations that became eradicated in the stochastic model, the population trajectories were similar to the deterministic models except that there was a larger difference in the abundance trajectories between culling patterns 2 and 3 (green and blue) in the deterministic models relative to the stochastic model over most of the trajectory (Fig 2, top). Also, for pattern 1, the stochastic model predicted higher abundances relative to the deterministic models over most of the trajectory until a large removal in the 4^th^ year drove the population to extinction. In general, the stochastic model also allowed for higher abundance during birth pulses relative to the deterministic models (Fig 2, top). In all three models, immigration severely limited the effects of culling on abundance (Fig 2, bottom). The deterministic models allowed for greater perturbations from culling patterns relative to the stochastic model (Fig 2, bottom). However, in the deterministic models with immigration, populations rapidly returned to carrying capacity whereas in the stochastic model, abundances remained lower than carrying capacity while culling was ongoing.

**Fig 3:**
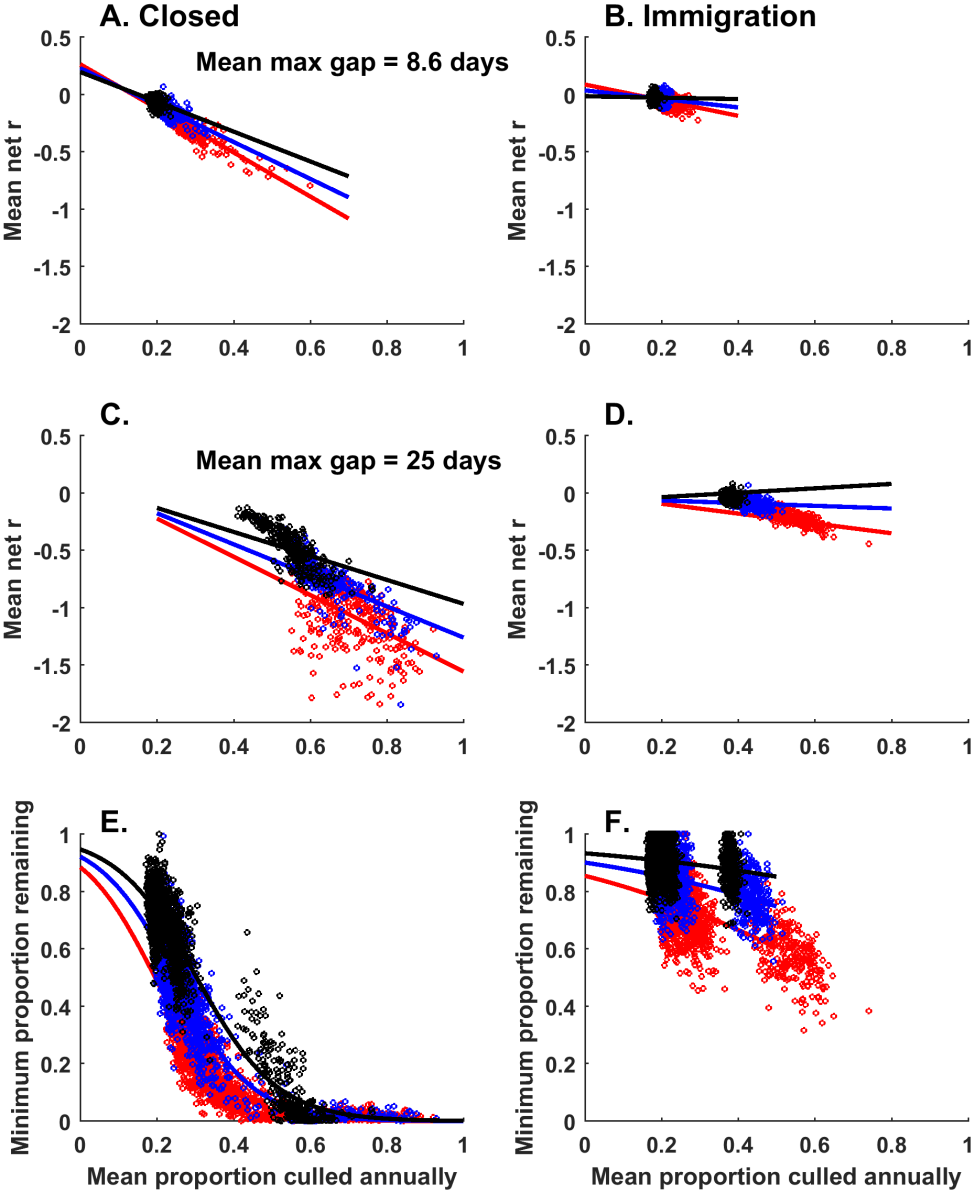
Relationship of population reduction metrics and culling conditions without (A,C,E) and with (B,D,F) immigration from surrounding areas. Points in A-D show all the data for the indicated culling intensity and gap period; points in E and F show all the data for all culling intensity and gap periods from similar conditions. Lines are predictions using the models presented in Tables A and B in S2 File. Colors indicate data from different intrinsic population growth rate parameters: Red: *r* = 0.26, Blue: *r* = 0.58, Black: *r* = 0.89. The A,B and C,D give predictions for two different gap lengths: 8.6 days (dotted), 25 days (solid). Predictions are cut-off to avoid predicting outside the range of the data.

Because population growth rates for wild pigs in the USA are largely unknown, we conducted all remaining analyses of culling patterns across 3 different intrinsic population growth rates (λ = 1.3, 1.78, and 2.43). We focused on the stochastic model because it represented wild pig biology most comprehensively. We averaged results across replicate simulations and other demographic conditions (maximum family group size and mean dispersal distance) because they were less influential in determining population dynamics. Intrinsic population growth rate, immigration, culling intensity, and gaps in culling were all important in determining a metric of culling efficacy: realized mean population growth rate (net *r*) in the first 4 years of culling (Table A in S2 File). There was an interaction between gap length (a metric of culling frequency) and culling intensity that obscured the main effects of these covariates on realized growth rate (Fig 3A–3D show effects of culling intensity on realized growth rates for two separate gap periods). As expected, realized growth rates decreased as culling intensity increased for different gap lengths, and the slope of this relationship increased with decreasing gap length indicating that realized growth rates were lowest when gap length was low but culling intensity was high (Fig 3A and 3C, Table A in S2 File). In the presence of immigration, the effects of culling intensity were very weak and only showed effects in the populations with low intrinsic *r* (Fig 3B and 3D). Within 4 years, populations could be reduced by > 95% with mean annual culling intensities of ~ 38%, 50%, and 60% in populations with intrinsic *r* = 0.26, 0.58, and 0.89; respectively (Fig 3E). For a 50% population reduction within 4 years, mean annual culling intensities of ~20–35% was required across the different intrinsic population growth rate conditions (Fig 3E). When immigration was allowed populations were never reduced more than 50%, which only occurred when the intrinsic population growth rate was low (*r* = 0.26), and the culling intensity was at the maximum we tested (60% annually, Fig 3F, Table B in S2 File).

**Fig 4:**
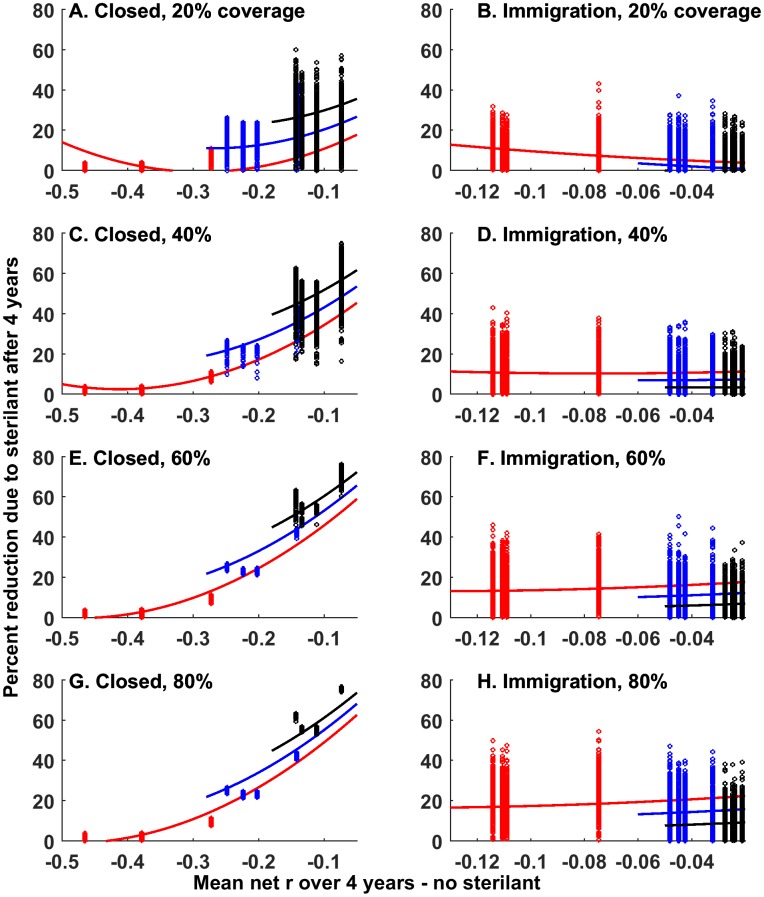
Effects of adding fertility control. Percent reduction in abundance due to sterilant (i.e., relative to culling only) after 4 years as a function of different net population growth rates (note: negative *r* values due to culling). Each point represents one simulation. Lines are predictions from the model presented in Table C in S2 File. Predictions were truncated outside the range of the data. Colors indicate data from different intrinsic population growth rate parameters: Red: *r* = 0.26, Blue: *r* = 0.58, Black: *r* = 0.89. A, C, E and G columns are for populations closed to immigration, B, D, F and H columns indicate scenarios where immigration from neighboring populations occurs. Predictions are cut-off to avoid predicting outside the range of the data.

The addition of fertility control, at levels of 40% of females annually (or greater), caused a substantially higher rate of population reduction (50–70% more reduction relative to culling alone) within 4 years in populations closed to immigration (Fig 4C, 4E and 4G, Table C in S2 File). This enhancement was highest in populations with the highest intrinsic population growth rates (*r* = 0.89), where culling alone was less effective. Enhancement was much weaker in populations with immigration, resulting in a maximum of ~30% enhancement with 40% coverage and 40% enhancement with 80% coverage (Fig 4F), although mean enhancement values were closer to 15% and 20%. In contrast to the populations with no immigration, populations with immigration showed the highest enhancement intrinsic populations growth rates were low (*r* = 0.26), and the amount of enhancement was similar across the full range of net *r* caused by culling in those populations (i.e., lines in Fig 4F are flat).

## Discussion

Efficient planning for controlling vertebrate pests depends on an understanding of population-dynamic processes and knowledge of demographic parameters. As we show here, knowledge of spatial and temporal processes that occur in wild pig populations and previously measured demographic parameters (references in Table 1) can be combined to predict the effectiveness of current patterns of wild pig culling (based on USDA activities). Incorporating data from realistic culling patterns helped us examine the effectiveness of a range of culling intensities and frequencies, which are implemented in practice, and assess whether fertility control could provide additional benefits under realistic culling patterns.

### Effects of culling intensity

A common question posed by wildlife managers is: “What annual harvest rate is needed to cause a decline or prevent population growth?” [53]. Considering the biology of wild pigs, we found that annual culling intensities of 20–60% of the population led to population declines (50–100% over 4 years) across a range of intrinsic population growth rates without immigration. With immigration, similar annual culling intensities led to an average population reduction of ~20% within 4 years (depending on intrinsic population growth rate) and the population maintained this lowered abundance, rather than continuing to decline. Our results are consistent with a previous analysis of hunter harvest data in Queensland, Australia (open to immigration) that found harvest rates as low as 20% could cause abundance reduction (30% over 4 years), as long as the harvest rates are maintained [54]. As the 30% abundance reduction is on the higher end of the range of our models with immigration (most realistic scenarios), it suggests immigration rates or intrinsic population growth rates in the Australian study may have been slightly lower than immigration rates in our model. It is likely that the immigration rates were lower in the Australian study because our low intrinsic population growth rates were quite low: 50% of field-based estimates (*r* = 0.26). Also, for immigration, we assumed that as soon as animals were culled, space was available to be occupied by dispersers or groups in cells at carrying capacity. However, in reality, there may be a lag time between these individuals seeking new space and the realization that the space is available, which could result in slower immigration than in our model.

Our result that annual culling intensities > 60% almost always led to > 99% reduction are also consistent with a previous modeling study based on demographic data from Texas, USA, (without immigration) that found that 40% annual culling intensities allow population growth while 66% leads to population declines [45]. However, in our model, culling intensities of 40% were also quite strong and led to population declines of 70–95% over 4 years in populations closed to immigration, while the previous work did not suggest such strong effects with 40% culling intensities. One reason for the discrepancy with [45] could be that our model included realistic lag times between conception and births, social structure, fluctuations around carrying capacity and spatial structure (these details were unclear in [45]).

Previous work suggests that the impacts of culling may be underestimated in deterministic models that neglect fluctuations around carrying capacity and lag times in reproductive processes [55]. When we scaled our deterministic models to have the same intrinsic population growth rates as our stochastic model, we found that the general patterns were similar between the two approaches but that the stochastic model tended to have larger abundances for slightly longer, and tended to persist longer. Thus, our results support Dexter & McLoed [55] because we found that the birth-pulse fluctuations in our stochastic model led to different effects of culling patterns on abundance trajectories relative to our deterministic models. But, in contrast to [55], we found that the populations with stochasticity tended to be more robust at low abundance because of the potential for higher fecundity at low densities relative to the deterministic models. The discrepancies between the simplified deterministic models and the more complex individual-based model suggest that deterministic model may be inappropriate for planning management in low-density populations.

### Effects of immigration on effectiveness of culling

As expected, intrinsic population growth rate affected the efficacy of control but the magnitude of intrinsic population growth rate effects depended strongly on immigration. In populations closed to immigration, where culling intensities were high enough to drive the population to zero under low intrinsic population growth rate, high intrinsic population growth rate prevented eradication. Alternatively, if immigration from surrounding areas was allowed, the outcome of different culling patterns was more similar across the different intrinsic population growth rates because immigration overwhelmed the effects of culling, and emphasizing that accurately predicting the effects of management depends on understanding immigration rates. Similarly, our statistical analysis predicted that culling intensity and gap length effects on abundance over time were weaker when immigration was allowed. Previous eradication efforts of wild pigs have found that blocking or severely reducing immigration into controlled areas (e.g., zoning by fencing) is critical to success of the program [11–14,56]. Our results predict that the importance of using fencing zones or other means of blocking immigration increases with intrinsic population growth rate. However, techniques such as fencing are only achievable for small-scale eradication programs, making it important to devise alternative strategies for national-scale control programs. Collecting data on population growth rates (abundance or density over time) alongside management, and combining this information with environment type covariate data and management activity maps may be a useful approach for predicting source populations that should be targeted for immigration control. Such predictions would inform spatial prioritization of culling (or other population management resources) by emphasizing where additional management should be conducted either at the border or within important source populations.

### Addition of fertility control

In agreement with previous work, we found that a sterilant used in conjunction with culling caused dramatically faster population decline under low culling intensities and high rates of fertility control [30]. We also found that even moderate levels of fertility control could provide benefits when used in conjunction with culling: the population was reduced 50% more relative to culling alone over a four-year period. This level of benefit required at least 40% of females be sterilized annually, which could be considered moderately high depending on the delivery mechnism of the sterilant. Thus our results suggest that moderately high (40% of females annually) levels of sterilization can accelerate population reduction in populations with ongoing culling [31]. However, our statistical analysis of the effects of net population growth rates on the efficacy of fertility control paints a more complicated picture than has been described previously [31], which suggests that the greatest response to fertility control will occur in populations that are declining, or have lower intrinsic population growth rate [31]. In contrast, we found that for closed populations, lower intrinsic population growth rate resulted in lower efficacy of fertility control relative to populations populations with higher intrinsic population growth rate. This was likely because simulated culling was intense enough in the populations with lower intrinsic population growth rate that fertility control could not provide additional benefits. This is consistent with the finding that fertility control provided no added benefits over poisoning in red foxes (*Vulpes vulpes*) [57]. Additionally, under the range of conditions we examined, we found that the populations without immigration and with low intrinsic population growth rate showed maximal effects of fertility control when net population growth rates were highest (near 0—we did not have data for conditions of net *r* > 0), but that effects of fertility control declined dramatically with net *r*. Thus, overall, our results show that a relativley narrow range of net population growth rates exist where application of fertility control in addition to culling may be beneficial—net *r* (the combination of intrinsic *r* and culling intensity) needs to be high enough that the population will not decline to eradication without additional control.

### Effects of immigration on effectiveness of fertility control

As has been noted previously [25,32,58], we found that immigration greatly reduced efficacy of fertility control. We found also that under conditions with immigration, intrinsic population growth rate had opposite effects on the efficacy of fertility control (relative to that in populations closed to immigration) along a similar gradient of population growth rate: fertility control was generally more effective in populations with lower intrinsic population growth rate (as would be predicted by [31] that did not consider immigration). This was because when immigration was allowed, the culling patterns were not strong enough to reduce abundance to low levels under any of the intrinsic population growth rate conditions, thus allowing fertility control to provide additional benefits. In cases with immigration, fertility control was only predicted to provide a maximum of 30% reduction with 40% coverage of females and 40% reduction with 80% coverage. Although this seems low, it is similar to the level of reduction provided by the culling patterns we examined under immigration. Thus our results show that addition of fertility control could almost double the magnitude of abundance reduction under immigration, suggesting that fertility control could be an important contribution to reduced abundance under conditions with immigration. One benefit that fertility control may have over culling under conditions with immigration and territoriality [40] or density dependence is that sterile individuals occupy areas that are unavailable for fertile immigrants (termed the ‘placeholder effect’ by [59]). This could serve as an important contribution to reduced abundance and immigration in controlled areas, buying time for additional immigration prevention techniques (e.g., culling at the border or within source populations) to be implemented. This placeholder effect method of maintaining lower abundance in target populations while culling source populations would act to maintain lower overall abundance in the target population, which could be particularly useful in target populations with high rates of immigration (i.e., where culling alone seems ineffective).

### Caveats

Although we explicitly modeled spatial locations and family group structure, the social and spatial ecology of wild pigs in our model only became relevant in our implementation of culling (i.e., targeting individuals in closest proximity to each other, thus removing whole family groups instead of random samples across groups). Therefore, our model does not account for territoriality [40] or other behavioral interactions in the probability that individuals will be sterilized or culled, meaning that our results in absolute terms are likely overestimates of population decline rates. We also simplified implementation of fertility control. We did not explicitly model the mating process, delivery or potential changes in behavior due to sterilants, which can affect the efficacy of fertility control [58,60–63]. We made these simplifying assumptions because we had no data for informing these processes, and also because we sought to understand the maximum potential effects of sterilants used in conjunction with lethal control. Previous work has also shown that fertility control tends to be more effective when density-dependence in the system acts through survival (as in our model), rather than through reproduction, which we did not include [64,65]. Thus, the realized effects of sterilants could be lower than our model predicts.

Although our analyses reveal important insight for implementation of control methods, two major gaps remain for accurate determination of optimal management strategies. For one, the relationship between damage levels and pig abundance is not well established in the USA. If we were to assume that the relationship is linear, then predicting the abundance reduction needed to reach a target level of damage would be straightforward. However, the relationship is likely more complicated such that at least some large reduction is needed in order to observe any effect on damage levels (i.e., culling patterns that do not meet some threshold of abundance reduction have no benefit to damage reduction) [66]. Thus, quantifying the relationship between abundance and damage is a critical ingredient to be considered in determining optimal strategies. Nonetheless, analyses without consideration of the damage-abundance relationship can still be enlightening when focused on the fastest reduction to eradication, as in some of our culling patterns, because these conditions would certainly eliminate damage. This is important because control techniques differ in efficacy according to abundance [13,23]. Studies designed to quantify the degree that effort increases as abundance decreases, as a function of multiple control techniques, is critical to predicting the most cost-effective solutions for minimizing abundance in target areas. Until these data become available, decision-making to reach a target level of damage should be based on previous experience of costs and effort for available techniques and knowledge of the effects of different strategies on abundance trajectories (as presented here, for example).

## Conclusions

As fiscal resource limitations are a major obstacle for large-scale wildlife management programs, it is important to carefully consider the efficacy of different practical control patterns on abundance reduction in order to prioritize resource allocation most effectively, and to evaluate the potential for new tools to enhance management outcomes. In wild pig populations closed to immigration, culling 20–60% of the population annually should result in rapid and substantial population reduction (50–100% within 4 years). However, with immigration, culling intensities must be at least 60% to reduce abundance by 50% over 5 years, which will only be possible when intrinsic population growth rates are on the low end for pigs (λ = 1.3). The addition of fertility control could provide additional reduction in abundance when used in conjunction with culling, but the amount of benefit depends strongly on demographic conditions and population growth rate as influenced by culling patterns. In summary:

- In conditions with culling and no immigration, fertility control will accelerate population decline when culling alone is slowing population growth but not drastically reducing abundance. In contrast, when intrinsic population growth rate is high enough that population growth continues despite culling, or when culling intensity is so high that culling alone reduces the population near 0 within 4 years, fertility control may not be worthwhile. Of course, in populations closed to immigration, the cost-effectiveness of simply increased culling should be weighed against that of using a combined strategy because, theoretically, simply increasing culling intensities should lead to eradication. Cost-effectiveness will be difficult to assess until a registered sterilant and delivery system are established.
- In conditions with immigration, effects of fertility control are weak (40% reduction in abundance at the most, 15–20% on average depending on conditions) but it provides benefits even when culling intensities are very high. Fertility control could be useful when immigration occurs because it may not be possible to reach target abundance with culling alone.

Success of control programs depends on rigorous monitoring programs alongside management in order to adaptively allocate resources and plan ongoing strategies in response to changing abundance [13,67]. Our results further emphasize that collecting demographic data such as fecundity and immigration rates could be important for adaptive allocation of resources. Collection of these and damage data alongside management will enable prediction of optimal resource allocation over space and time.

## Supporting information

S1 File Supplementary figures. (PDF)

S2 File Additional statistical methods and results. (DOCX)

S3 File MIS culling data. Temporal sequence of number of pigs culled for each of the five properties and the control (i.e., all zeros because there was no culling).
(CSV)
