# Supplementary material for: Potential effects of incorporating fertility control into typical culling regimes in wild pig populations
Source: PLoS One. 2017 Aug 24;12(8):e0183441. doi: 10.1371/journal.pone.0183441 (PMC5570275; doi:10.1371/journal.pone.0183441)
Supplement: S1 File — (PDF) [file pone.0183441.s001.pdf]

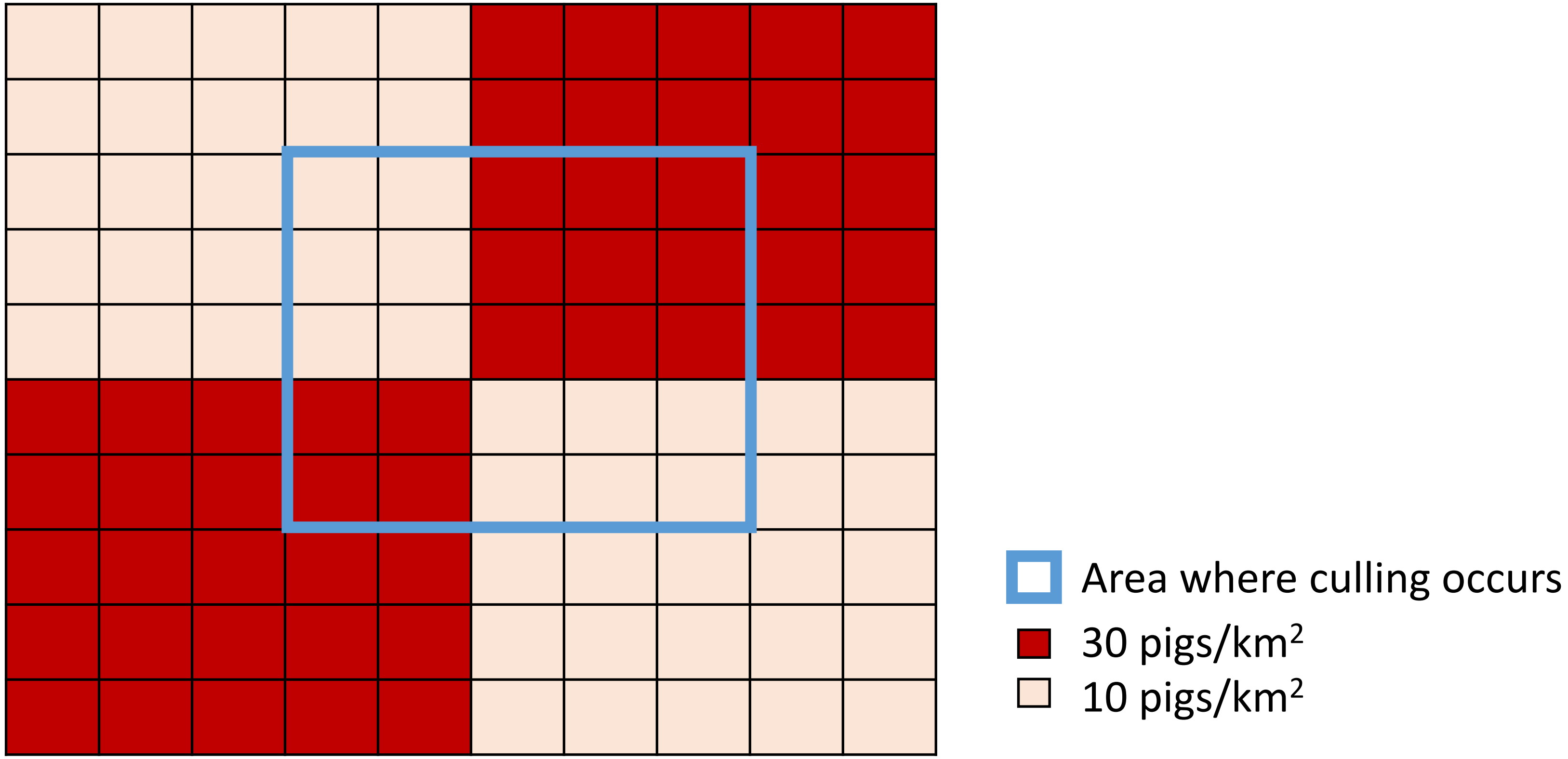

Figure A. Design of landscape. Each cell is 1 km^2. The blue square indicate where culling was confined. Pigs existing outside the blue square are available for immigration into the blue square area as space becomes available. Colors indicate different maximum pig densities.

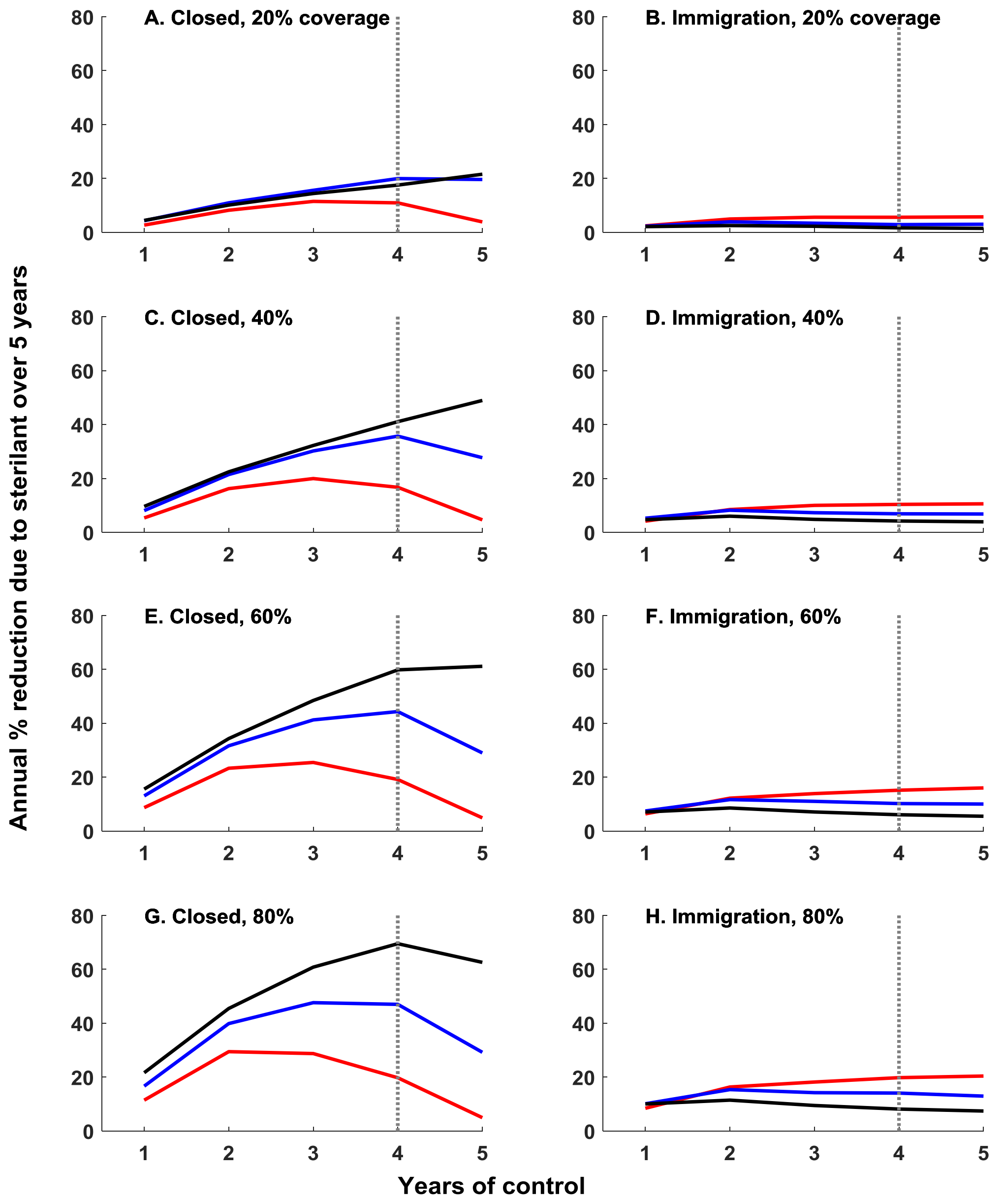

Figure B. The additional advantage of adding fertility control in addition to culling during each year of control. Y-axis is the % reduction in abundance due to sterilant (i.e., relative to culling only) as a function of different intrinsic population growth rates: low (red), field-based (blue), high (black). A, C, E and G columns are closed to immigration, B, D, F and H columns indicate scenarios where immigration from neighboring populations occurs. Each line is the mean of 270 replicates. The grey vertical dotted line shows the time point that analyses from Tables S1-S3 and Figures 5 and 6 were conducted on.

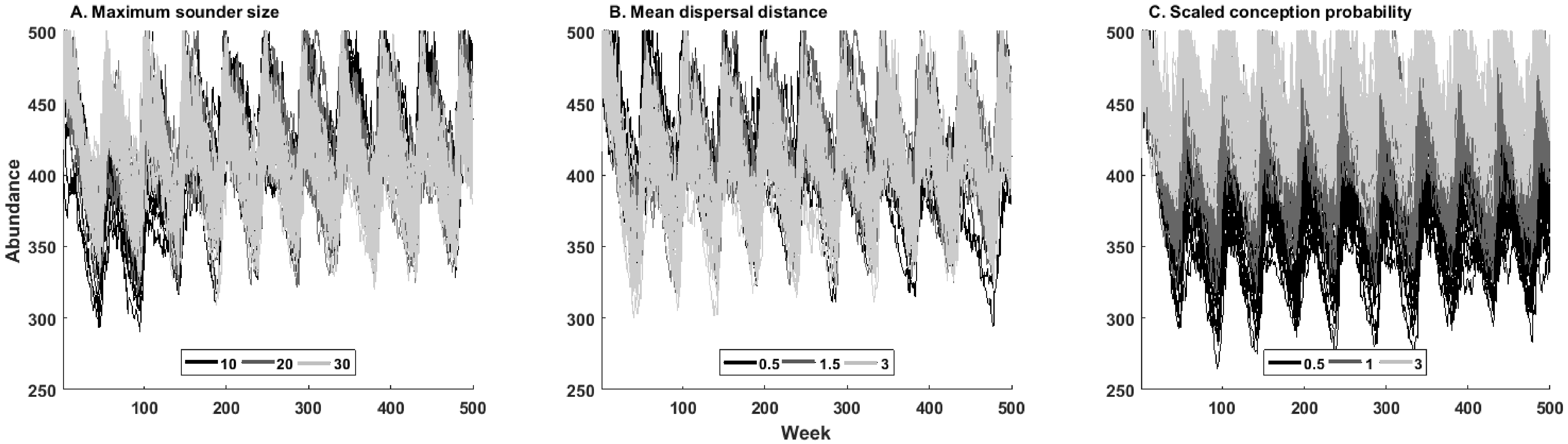

Figure C. Population dynamics with no culling or immigration. Effects of demographic parameters (A-C) on weekly abundance over 10 years. A. Maximum numbers of pigs per family group. B. Mean dispersal distance in km (~EXP(ξ km)). C. Scaling factor on weekly conception probability (where weekly conception probability is multiplied by this factor to scale the birth rates up or down). Legends indicate the 3 separate demographic conditions shown in the plots. All 30 stochastic replicates for each set of conditions are shown. Within each plot, other demographic parameters were held constant (i.e., variation is only from stochasticity) at fixed values: 20 for maximum family group size, 1.5 for mean dispersal distance and 1 ('field-based') for scaling on conception probability (intrinsic *r*). Results show that realistic variation in maximum group size and dispersal distance did not affect population dynamics – stochastic differences introduced as much variation as these parameters (A and B). In contrast, conception probability strongly determined population dynamics with the highest value leading to the highest abundance over time, and the differences appear substantial despite stochastic variation.

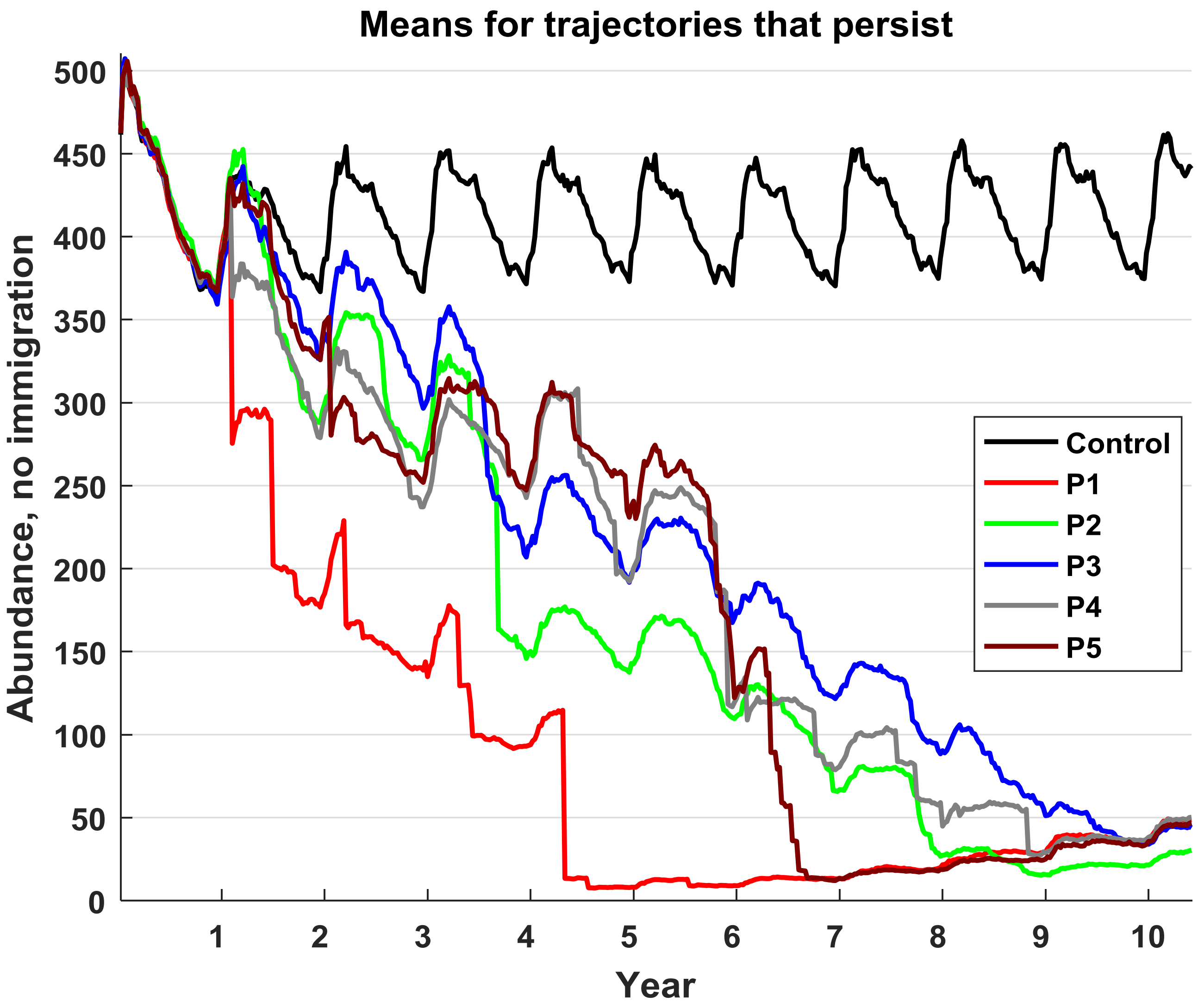

Figure D. Same as Figure 2A, top except these are means of the trajectories that did not become eradicated.
