# Supplementary material for: Potential effects of incorporating fertility control into typical culling regimes in wild pig populations
Source: PLoS One. 2017 Aug 24;12(8):e0183441. doi: 10.1371/journal.pone.0183441 (PMC5570275; doi:10.1371/journal.pone.0183441)
Supplement: S2 File — (DOCX) [file pone.0183441.s002.docx]

**Supporting Information - Methods for:**

**“Potential effects of combined management strategies on controlling wild pig populations”**

**Kim M. Pepin, Amy J. Davis, Fred L. Cunningham, Kurt C. VerCauteren, Doug C. Eckery**

**Supplemental Methods: Statistical Analyses**

To help visualize the relationships between factors driving the population trajectories, we constructed statistical models of the simulated data using all the ecological and management factors we were interested in. Management factors included culling intensity (Pcull), gap periods between sequential culling events (gap), and the percent of the population sterilized (C). Ecological factors included intrinsic rate of population increase (intrinsic *r*), net rate of population increase (net *r*), and immigration status (allowed or not) – all defined in more detail below. We were interested in how three different responses were affected by these covariates:

1) The average net annual population growth rate (net *r*) over the first 4 years of culling (Table A below). For this, we were interested in the effects of culling patterns (intensity and gap periods) and demographic conditions (intrinsic *r* and immigration status) on realized population growth rates. We calculated mean net *r* as:

$net r= \frac{\sum_{t=1}^{T} log\left( \frac{N_{t}}{N_{0}} \right)}{T}$, where *T* = 4 years and *N* was from the last week of year *t*.

2) The minimum proportion of the population remaining after 4 years of culling (Table B below). For this we were interested in the same parameters as in 1) (except for gap period) on the magnitude to which the population could be reduced over 4 years. We calculated the proportion remaining as: N_t_/N_0_ where N_0_ was the initial abundance in the week before *any* culling began (i.e., last week of the burn-in period) and N_t_ was the abundance in the last week of year *t*, and t = 1,2,3, or 4. We then took the minimum value over the 4 years.

3) The difference in the proportion by which the population is reduced after 4 years (as calculated in 2)) in populations without and with fertility control. For this response, we were interested in the net *r* due to culling and coverage of fertility control, and the demographic effects of intrinsic *r* and immigration status.

We chose 4 years as the time frame to examine effects because it is a reasonable amount of time to expect strong management outcomes and because plots of management effects over time (Fig 2 and Fig B in File S1) showed this to be the time frame to observe the strongest effects across the widest range of conditions (Fig B in File S1). The full models used for prediction are specified in their respective table captions. We verified that all parameters in the model were informative using AIC. To better understand the interplay of the management and demographic factors we were interested in, we predicted from the models. To verify the predictive ability of our models we performed 10-fold cross-validation by: randomly selecting 10% of the data to be withheld (test set), fitting the model to the remaining 90% (training set), and predicting the test set from parameters estimated from the training set. We repeated this procedure 100 times on different randomly sampled test sets and calculated the mean and standard deviation of the test set scores. Both mean squared errors and adjusted R^2^ for the test data were similar to those of the training data (see below each respective table).

**Explanation of parameter names**

**Intrinsic *r*:** Intrinsic reproductive rate of the population (continuous variable with 3 possibilities: 0.26, 0.58, 0.89 which are equivalent to λ = 1.3, 1.78, and 2.43, and our conception probability scaling parameter of 0.5, 1, 3; respectively)

**Net *r*:** Mean annual population growth rate (realized) for the first 4 years of culling.

**I:** Immigration (factor with 2 levels: yes or no)

**Pcull:** Culling intensity - mean annual proportion culled in the first 4 years, where proportion culled annually = actual number culled in year *t* (i.e., αc) / mean abundance in year t-1 (continuous variable). We took the average over 4 years (as opposed to just an overall fraction) because the proportion culled depends both on the number removed and the current abundance which is changing in time non-linearly. Thus, the average is a better overall measure of culling intensity over the whole trajectory.

**gap:** Mean of maximum number of weeks between culling events during the first 4 years (integer)

**C:** Coverage of the sterilant (factor with 4 levels: 20%, 40%, 60%, 80%)

**Table A.** Parameters of the model used for prediction in Figure 3 (top and middle). The model included: Pcull, Intrinsic *r,* Immigration (factor), and gap as main effects with all 2-way interactions between them. The response was the mean annual population growth during the first 4 years of control. Adjusted R^2^ for the full fit = 0.92. Data were modeled using a Gaussian distribution.

Cross-validation statistics:

(Mean of 100 bootstrapped replicates ± 1 standard deviation)

R^2^ of training data: 0.917 +/- 0.0011

MSE of training data: 0.0055 +/- 0.00010

R^2^ of out-of-sample predictions: 0.916 +/- 0.0099

MSE of out-of-sample predictions: 0.0055 +/- 0.00095

**Table B.** Parameters of the model used for prediction in Figure 3 (bottom). The model included: Pcull, Intrinsic *r,* and Immigration (factor) as main effects with all 2-way interactions between them. The response was the minimum proportion of the population remaining after 4 years of control. Adjusted R^2^ for the full fit = 0.94. Data were modeled using a binomial distribution and logit link.

Cross-validation statistics:

(Mean of 100 bootstrapped replicates ± 1 standard deviation)

R^2^ of training data: 0.935 +/- 0.0005

MSE of training data: 0.0059 +/- 0.000034

R^2^ of out-of-sample predictions: 0.935 +/- 0.0044

MSE of out-of-sample predictions: 0.006 +/- 0.0003

**Table C.** Parameters of the model used for prediction in Figure 4. The model included: Net *r*, Intrinsic *r*, Immigration (factor), and Sterilant coverage (factor) as main effects with all 2-way interactions between them. The model also included a squared term for net r because we expected this relationship to be non-linear and the term was supported using AIC. The response was the reduction in pig population due to sterilant after 4 years of control (calculated as y = proportion remaining (culling only) - proportion remaining (culling + sterilant)). Adjusted R^2^ for the full fit = 0.79. Data were modeled using a Gaussian distribution.

Cross-validation statistics:

(Mean of 100 bootstrapped replicated ± 1 standard deviation)

R^2^ of training data: 0.790 +/- 0.0009

MSE of training data: 0.008 +/- 0.000027

R^2^ of out-of-sample predictions: 0.788 +/- 0.0083

MSE of out-of-sample predictions: 0.0081 +/- 0.00024
